# Multiplex PCR System for the Diagnosis of Plague

**DOI:** 10.2174/0115665240321812240918095931

**Published:** 2024-09-24

**Authors:** Wenfang Wang, Xiaoxue Zhang, Hui Yu, Zhanli Wang

**Affiliations:** 1 Inner Mongolia Key Laboratory of Disease-Related Biomarkers, The Second Affiliated Hospital, Baotou Medical College, Baotou, China;; 2 School of Basic Medicine, Baotou Medical College, Baotou, China

**Keywords:** Multiplex PCR, plague, *Yersinia pestis*, fatality rate, mosquito bites, zoonotic bacteria

## Abstract

The plague caused by *Yersinia pestis* has a high case fatality rate. It is often transmitted from person to person through mosquito bites, causing serious disease transmission. Due to its clinical symptoms being very similar to influenza, it is difficult to detect by people. Traditional detection methods for *Y. pestis* mainly include bacterial culture and serological identification, which are cumbersome and require high experimental conditions. Therefore, a fast and effective detection method is very important. At present, polymerase chain reaction (PCR) is one of the methods for rapid detection of *Y. pestis*. In this review, we focus on the application, advantages, and disadvantages of multiplex PCR technology in clinical detection.

## 1 INTRODUCTION

Plague, also known as the Black Death, is a virulent infectious disease caused by *Yersinia pestis* (*Y. pestis*). For centuries, it has been considered a unique disease entity by doctors and people because it is the only disease characterized by enlarged lymph nodes (known as inguinal lymphadenitis) that can cause deadly epidemics [1]. In history, there have been three major outbreaks of plague, which have spread widely and seriously affected tens of millions of people [2]. The first pandemic occurred in the 6^th^ century AD and ravaged the Mediterranean basin. The Black Death in Europe in the 14^th^ century killed up to 30% of the population. The latest pandemic began in Asia and India in the late 19^th^ century and continues to spread in Africa today [3, 4]. Recently, *Y. pestis* has received attention as a bioterrorist agent [5-7], posing a serious threat to human life and health [8, 9].

*Y. pestis* belongs to the Enterobacteriaceae family [10]. It is a gram-negative, non-motile, non-spore-forming coccidia (0.5 to 0.8 μm in diameter and 1 to 3 μm in length). Polarity staining is demonstrated by Giemsa, Wright, or Wayson staining. It was first isolated by Alexander Yersinia during the third plague pandemic in Hong Kong. In addition to *Y. pestis*, the genus Yersinia also includes *Yersinia pseudo-tuberculosis (Y. pseudotuberculosis)* and *Yersinia enterocolitica (Y. enterocolitica)*, which are also associated with human infection and cause mild diarrhea [11]. *Y. pestis* is highly similar to the enteric pathogen *Y. pseudotuberculosis* at the genomic level, and the acquisition and deletion of a series of genes have resulted in *Y. pestis* exhibiting different pathogenic mechanisms and cultural characteristics [12]. Notably, *Y. pestis* has specific virulence factors that can successfully infect fleas and disrupt the mammalian immune system, leading to rapid host death in the absence of proper treatment [13, 14].

**Fig. (1):**
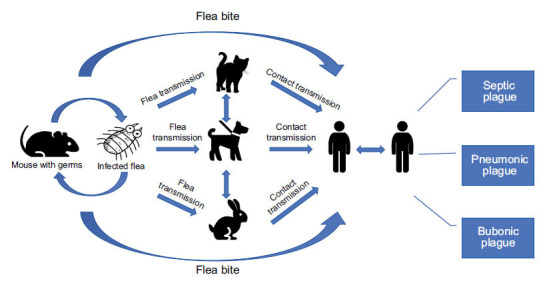
Routes of plague transmission. Flea bites are the main routes of transmission, and some can also be transmitted through person-to-person contact.

Currently, plague is one of the most important bacterial zoonotic diseases in the world [15, 16]. It occurs naturally in wild rodents, which act as reservoirs of the bacteria [17]. Other small carnivorous animals, such as wild dogs, rabbits, and cats, can also act as carriers and become sources of human infection [18, 19]. The most common source of infection for plague is infected fleas. When fleas feed on infected rodents, individual planktonic bacteria enter the midgut, and some fleas excrete all *Y. pestis* through defecation. *Y. pestis* sometimes cannot be discharged completely and continues to parasitize in fleas [20]. When humans come into contact with contaminated fleas, there is a risk of infection [21]. Moreover, *Y. pestis* infection might occur through ingestion of contaminated food or water, physical contact with infected patients, or direct inhalation of infectious respiratory droplets (Fig. **1**) [22].

There are three main clinical forms of *Y. pestis* infection in humans: bubonic, septicemic, and pneumonic plague [23]. Generally, three to seven days after the incubation period, infected people usually begin to exhibit “influenza-like” symptoms, with clinical manifestations mainly including high fever, lymph node swelling and pain, bleeding, lung inflammation, and so on [24, 25]. In cases of the Black Death, patients typically experience localized redness, dryness, and burning of the skin, progressive severe pain at the site of flea bites, and intense pain due to swollen lymph nodes leading to forced positions [26, 27]. Then, it quickly develops into a purulent, foamy cough or, eventually, a bloody cough. Chest X-ray examination of pneumonic plague may show lobar pneumonia, which will quickly spread throughout the lung. In addition, bloody sputum has strong infectivity.

Traditional detection methods for *Y. pestis* mainly include bacterial culture and serological testing [28]. The gold standard for diagnosing epidemics is bacterial culture [29]. However, the transportation of specimens is difficult, which may lead to bacterial contamination or even death. Meanwhile, the experimental process is time-consuming, delaying timely treatment for patients, and the results are prone to false negatives. The operating technology of the inspectors, the high-quality culture environment, and the influence of external conditions are the main reasons for the low sensitivity of the bacterial culture method. Serological testing can also be used to detect whether a person is infected with *Y. pestis*. However, there may be delayed antibody reactions after contact, and previous contact may lead to positive results [30, 31]. These methods may be time-consuming, complex to operate, less sensitive, and require specialized laboratories and trained technicians [32]. Therefore, it is necessary to develop a simple and rapid detection method with high sensitivity and good specificity. It helps to detect *Y. pestis* quickly and take preventive measures in time. In addition, early diagnosis and treatment can effectively reduce the mortality of bubonic and septicemic plague [33, 34].

## 2 TARGET GENES OF *Y. PESTIS* FOR PCR AMPLIFICATION

Mullis *et al.* designed a new method that can directly amplify nucleic acid sequences *in vitro* – PCR [35]. A thermostable DNA polymerase is used to produce a copy of the DNA sequence, and the annealed flanking sequence is amplified by lengthening two oligonucleotides [36]. PCR is widely used in many fields and is a routine technique in clinical diagnosis. It has been shown that PCR technology can be used to detect very small amounts of microorganisms by amplifying target sequences [37]. Compared with the traditional methods mentioned above, the application of PCR technology to detect *Y. pestis* only takes about four hours, and the experimental steps are simple, which greatly shortens the detection time and provides valuable time for clinical diagnosis and treatment of patients. At present, PCR has become a new method to detect *Y. pestis*, with high sensitivity and specificity [38, 39]. Engelthaler *et al.* reported that PCR detection technology has high sensitivity and specificity [40]. For *Y. pestis*, the commonly used PCR target genes are chromosome genes and plasmids. For example, Pestering (*pst*), plasminogen activator protease (*pla*), 60-Md plasmid-located gene(*caf1*), invasin protein gene (*inv*), and so on, among which the most common target groups are *pla* and *caf1* [41-45].

*Y. pestis* generally has three classical plasmids. The smallest 9.5 kb pPCP and the largest 100 kb pMT are unique to *Y. pestis*, and the medium-sized 70 kb pCD plasmid is homologous to the plasmids in *Y. pseudotuberculosis* and *Y. enterocolitica*, two other human Yersinia pathogens [46, 47]. The virulence determinant is encoded on all three plasmids and chromosomes of *Y. pestis*. In animal models, the absence of these plasmids can have different effects on virulence, leading to the emergence of the *Y. pestis* subtype in nature [48, 49].

The *pla* encoded by 9.5-kb small plasmids is a member of the omptin family, which is composed of outer membrane proteins classified as bacterial aspartate proteases and is a multifunctional protein [50]. It may function by interfering with the speed's ability to control infection locally. In particular, *Y. pestis* can use coagulase activity to produce fibrin clots that block the digestive tract of fleas. This process increases the survival rate of *Y. pestis*, so the possibility of the bacteria spreading through fleas increases [51, 52]. In addition, it is believed that it can promote the spread of bacteria from the main infection site and may be responsible for the highly invasive and explosive characteristics of *Y. pestis* infection [53, 54]. Furthermore, it was believed for a long time that it is absent from closely related Yersinia species. However, the *pla* gene has also been detected in *Citrobacter koseri*, *Escherichia coli* [55, 56]. Therefore, it is necessary to develop multiplex PCR for detecting *Y. pestis* with *pla* and other target genes [57].

 Moreover, pFra harbors the *ymt* gene, which displays phospholipase D activity. It is involved in the metabolic adaptation of *Y. pestis* to the flea gut, which is necessary for the survival of *Y. pestis* [58]. It also encodes a fimbrial protein that accumulates on the bacterial surface to form an amorphous capsule [59, 60], which is known as Fraction I or F [61]. *Y. pestis* expresses a unique capsule-like F1 antigen at 37°C [62]. The F1 antigen provides *Y. pestis* with the ability to block phagocytosis through a different mechanism than T3SS and pH 6 antigen [63]. *Caf1* is one of the most expressed genes during mammalian infection [64].

In addition to plasmids, chromosome genes can also be used as target genes. The ability of bacteria to invade cells outside the body is mainly due to a chromosomal gene called *inv* [65]. Southern blot analysis proved the existence of *inv*-related sequences in *Y. pestis* [66]. The *inv* gene is also often used as a target gene to detect *Y. pestis*.

The above plasmids can be detected in *Y. pestis* with typical characteristics, but it is reported that *Y. pestis* with one or more plasmids exists in nature [67, 68]. It was reported that in 1960, a Fl deficient strain had been identified from deadly human cases of plague. Williams *et al.* also isolated *Y. pestis* derivatives from plague patients, rodents, and fleas that lack one or several virulence plasmids. This atypical type of bacteria will lead to fatal or mild plague cases, as well as chronic and recessive infections [69, 70]. Due to the absence of plasmids, PCR detection methods based on a single target gene may result in false negative results. Higgins *et al.* found that the PCR detection technology only targeting the *Pla* gene could not identify the atypical *Y. pestis* with *Pla* plasmid deletion caused by mutation, which may result in false negative results [71]. With the deepening of PCR technology research, many PCR methods have been used to detect *Y. pestis*, including conventional PCR [72, 73], multiplex PCR [74, 75], real-time fluorescent quantitative PCR [76, 77], nested PCR [78, 79] and other technologies. In this review, we describe the detection of *Y. pestis* by multiplex PCR technology.

## 3 MULTIPLEX PCR

Multiplex PCR is a technique in which more than one target gene is amplified simultaneously in a reaction system. It can improve the accuracy of detection. Leal *et al.* developed a multiplex PCR method using multiple pairs of primers, which has been shown to distinguish strains lacking one or more known pathogenic loci [80]. Riehm *et al.* reported the development of an internal real-time PCR detection for three typical plasmid genes of *Y. pestis*, and the accuracy rate is higher than that of bacterial culture and ITC detection [81]. Stewart *et al.* developed an assay that could quickly and accurately identify *Y. pestis* isolates [82].

Additionally, they think it has the potential to complement a diagnostic tool for rapid identification of *Y. pestis*. Matero *et al.* developed a multiplex PCR detection technology to distinguish *Y. pseudotuberculosis* and *Y. pestis* [83]. The detection limit of this method is 10 – 100 fg of the total number of *Y. pseudotuberculosis* or *Y. pestis* extracted. Table **1** shows the primers used in multiplex PCR.

There are currently multiple possible biological weapon pathogens, and rapid identification of pathogens is crucial, with the aim of initiating specific treatment and control measures [84]. Multiplex PCR detection technology can be used to simultaneously identify multiple pathogens [85, 86], which may save time, quickly diagnose diseases, and make correct diagnosis and treatment plans [87]. Kuske *et al.* developed a multiplex PCR technique for the simultaneous detection of four bacteria: *Bacillus anthracis*, *Clostridium perfringens*, *Y. pestis*, and *Francisella tularensis* [88]. The experiment exhibited good sensitivity and specificity, and the LOD of four target multiplex assays was determined to be 1 CFU per reaction. Similarly, Skottman *et al.* developed a multiplex PCR assay that can simultaneously detect *B. anthracis*, *F. tularensis,* and *Y. pestis*, also with high sensitivity and specificity [89]. Yang *et al.* designed the pathogens-specific TaqMan probes for simultaneous detection of *B. anthracis*, *Y. pestis*, and *F. tularensis* [90]. These methods can detect both common bacteria and highly dangerous pathogens, providing timely guidance for clinical treatment and improving detection efficiency.

 Multiplex PCR techniques are currently used to detect *Y. pestis*, typically using total DNA obtained from bacterial cultures and human peripheral blood as a template to distinguish strains that lack one or more known disease-causing sites. In multiplex PCR reaction systems, due to simultaneous amplification of multiple primers, cross-pairing, and accumulation of primers may occur. Therefore, the optimization of the multiplex PCR reaction system is particularly important. Under normal circumstances: predenaturation 95°C, 5 min, denaturation 95°C, 30s, annealing 55°C, 30s, extension 72°C, 45s, 40 cycles. However, in the course of the experiment, appropriate adjustments need to be made according to the reagent used, the size of the target fragment, the Tm value of the primer, etc. After amplification, the amplified products were electrophoretic and used for presumptive identification of members of *Y. pestis*. This assay can also evaluate the robustness of PCR technology and whether the corresponding bands can be accurately amplified.

PCR technology is highly sensitive. Due to the small amount of PCR reaction system, small changes in the operation process will affect the experimental results. Therefore, contamination and minor errors during operation are the main reasons that affect the sensitivity of PCR reaction. The specificity of PCR techniques is influenced by a number of factors, including annealing time, temperature, elongation time, primer design, and enzyme concentration. For example, increasing the annealing temperature can reduce non-specific amplification and thus improve specificity; Reducing primer and enzyme concentration can reduce primer dimerization. In reported studies, it has been shown that multiplex PCR for the detection of *Y. pestis* can improve the specificity of the method without reducing sensitivity compared to traditional PCR. At the same time, it also shows that the multiplex PCR technique has good clinical application value and can be used as a rapid diagnostic method for the diagnosis of *Y. pestis*.

Moreover, multiplex PCR can combine with other techniques to screen multiplex pathogens at the same time. Elsholz *et al.* developed a new type of electric low-density microarray based on multiple PCR assays, which can simultaneously detect four pathogenic microorganisms, including *B. anthracis*, *Y. pestis*, *F. tularensis,* and *Orthopoxvirus* [91]. The electrical microarray was shown to be sensitive enough to analyze very low PCR products. Regan *et al.* developed a multiplex PCR amplification combined with a microsphere array detection technique with high specificity and reliability [92]. Wilson *et al.* developed a 10-plexed PCR assay coupled to a 12-plexed liquid bead array to rapidly screen environmental samples of *B. anthracis*, *Y. pestis*, *F. tularensis*, and *B. melitensis* [93]. Deshpande *et al.* developed a rapid (under 4 hours), multiplex, nucleic acid assay adapted to a microsphere array detection platform and applied it to the detection of three biothreat agents, such as *B. anthracis*, *Y. pestis*, and *F. tularensis* [94]. Ii *et al.* found that CN3080 Nanotraps could bind tightly to *Y. pestis*, which enhanced the ability of PCR to detect *Y. pesis* [95]. Previous studies also reported that multiplex PCR can be used to detect DNA, conform pathogenic bacteria, or transmit transmission routes [96, 97].

## CONCLUSION

For some zoonotic bacteria that cause serious diseases in humans, they may be used for bioterrorism attacks [98-100]. Therefore, rapid and reliable detection of these organisms is essential for rapid and effective patient management and disease control [101]. Multiplex PCR technology has the advantages of simplicity, rapidity, high sensitivity, and specificity. The application of multiplex PCR technology in targeted pathogen detection has significantly improved the efficiency and accuracy of pathogen detection. Multiplex PCR technology can also detect multiple pathogens at the same time, which can be used for routine monitoring of the environment to prevent the spread of *Y. pestis* and other pathogens. In addition, with the continuous progress and innovation of multiplex PCR technology, such as the increase of detection flux, the improvement of detection rate, and the continuous reduction of cost, the application prospects of multiplex PCR technology will continue to expand. However, PCR inhibitors may exist in the samples. The laboratory conditions and DNA extraction quality also directly affect the sensitivity and specificity of multiplex PCR methods. In addition, the multiplex PCR reaction system requires an appropriate temperature to reduce non-specific expansion. Therefore, future research should focus on system optimization and DNA extraction. Although the detection of *Y. pestis* by multiplex PCR technology cannot be used as a standard for the independent diagnosis of plague in the clinic, it is believed that with the deepening of PCR technology, this method will be widely used in the diagnosis of *Y. pestis* and play an irreplaceable role.

## Figures and Tables

**Table 1:** Sequences of primers for the multiplex PCR.

**Genes**	**Primer Sequences (5’→3’)**	**Product**	**Location**	**References**
*Caf1*	Forward CAGTTCCGTTATCGCCATTGC	501	pMT1	[36]
	Reverse TATTGGTTAGATACGGTTACGGT		-	
*Pla*	Forward TGGATGAATGAAAATCAATCTGAG	443	pPCP1	
	Reverse ATAATATCCAGCGTTAATTACGGT		-	
*Caf1*	Forward CAGGAACCACTAGCACATC	171	pMT1	[70]
	Reverse CCCCCACAAGGTTCTCAC		-	
*Inv*	Forward TAAGGGTACTATCGCGGCGGA	295	chromosome	
	Reverse CGTGAAATTAACCGTCACACT		-	
*Pla*	Forward ATCTTACTTTCCGTGAGAAG	480	pPCP1	
	Reverse CTTGGATGTTGAGCTTCCTA		-	
*YopM*	Forward ATAACTCATCGGGGGCAAAAT	565	chromosome	
	Reverse GCGTTATTTATCCGAATTTAGC		-	
*Caf1*	Forward GGGAATTCCAGGTAATATATGAAAAAAATCA	506	pMT1	[80]
	Reverse CCGCTGCAGATTATTGGTTAGATACGG		-	
*LcrV*	Forward AGAGCCTACGAACAAAACCCAC	800	pYV	
	Reverse GCAGGTGGTGGCAAAGTGAGA		-	
*Pla*	Forward AAGTTCTATTGTGGCAACC	920	pPCP1	
	Reverse GAAGCGATATTGCAGACC		-	
*Irp2*	Forward AAGGATTCGCTGTTACCGGAC	300	chromosome	
	Reverse TCGTCGGGCAGCGTTTCTTCT		-	
*YopT*	Forward GATCAGGAGCCATGCACAA	330	pCD1	[82]
	Reverse ACATTTGGCCTGAGAGATGTA		-	
*VirF*	Forward GGCAGAACAGCAGTCAGACATA	561	pYV1	
	Reverse GGTGAGCATAGAGAATACGTCG		-	
*Ymt*	Forward AGGACCTAATATGGAGCATGAC	168	pMT1	
	Reverse CTAACAAAGCCTCAATAATCCA		-	
*Pst*	Forward GAATGGTTCAGGTGGTGTTCC	2159	pPCP1	
	Reverse TTCTCCATCTCCGTATCAATCG		-	

## ABBREVIATION

PCR: Polymerase Chain Reaction
